# Different mechanosensory stimulations of the lower back elicit specific changes in hemodynamics and oxygenation in cortical sensorimotor areas—A fNIRS study

**DOI:** 10.1002/brb3.575

**Published:** 2016-10-14

**Authors:** Andrea Vrana, Michael L. Meier, Sabina Hotz‐Boendermaker, Barry K. Humphreys, Felix Scholkmann

**Affiliations:** ^1^ Interdisciplinary Spinal ResearchDepartment of Chiropractic MedicineUniversity Hospital of BalgristZurichSwitzerland; ^2^ Department of Health Sciences and Technology, Human Movement SciencesETH ZurichZurichSwitzerland; ^3^ Biomedical Optics Research Laboratory (BORL)Department of NeonatologyUniversity Hospital ZurichUniversity of ZurichZurichSwitzerland

**Keywords:** back pain, functional near‐infrared spectroscopy, hemodynamic response, sensorimotor cortex

## Abstract

**Background and Objectives:**

This study aimed at investigating the feasibility of functional near‐infrared spectroscopy (fNIRS) to measure changes in cerebral hemodynamics and oxygenation evoked by painful and nonpainful mechanosensory stimulation on the lower back. The main objectives were to investigate whether cortical activity can be (1) detected using functional fNIRS, and (2) if it is possible to distinguish between painful and nonpainful pressure as well as a tactile brushing stimulus based on relative changes in oxy‐ and deoxyhemoglobin ([O_2_Hb] and [HHb]).

**Methods:**

Twenty right‐handed subjects (33.5 ± 10.7 years; range 20–61 years; 8 women) participated in the study. Painful and nonpainful pressure stimulation was exerted with a thumb grip perpendicularly to the *spinous process* of the lumbar spine. Tactile stimulation was realized by a one‐finger brushing. The supplementary motor area (SMA) and primary somatosensory cortex (S1) were measured bilaterally using a multichannel continuous‐wave fNIRS imaging system.

**Results:**

Characteristic relative changes in [O_2_Hb] in the SMA and S1 after both pressure stimulations (corrected for multiple comparison) were observed. [HHb] showed only much weaker changes (uncorrected). The brushing stimulus did not reveal any significant changes in [O_2_Hb] or [HHb].

**Conclusion:**

The results indicate that fNIRS is sensitive enough to detect varying hemodynamic responses to different types of mechanosensory stimulation. The acquired data will serve as a foundation for further investigations in patients with chronic lower back pain. The future aim is to disentangle possible maladaptive neuroplastic changes in sensorimotor areas during painful and nonpainful lower back stimulations based on fNIRS neuroimaging.

## 1 Introduction

Lower back pain (LBP) has life‐time prevalence up to 85% (Andersson, 1997; Balague, Mannion, Pellise, & Cedraschi, 2012) worldwide. In Switzerland, a large population survey revealed that 47% of women and 39% of men suffered from back pain in the preceding 4 weeks (Bundesamt für Statistik 2007). The International Association for the Study of Pain (IASP) defines pain as “*an unpleasant sensory and emotional experience associated with actual or potential tissue damage, or described in terms of such a damage*” (Loeser & Treede, 2008). Typically, the majority of acute low back pain patients recover within a few days or weeks, although a small minority of around 5–10% of patients become chronic (i.e., pain lasts >3 months) (Andersson, 1999). In patients with chronic pain, pain loses its preventive function, and becomes severely disabling and accompanies patients mostly throughout life (Andersson, 1999; Blumer & Heilbronn, 1982; Brown, 1990). The main reason for a lack of applicable treatment methods lies mainly in the lack of specific knowledge about how the multidimensional pain experience is processed within the central nervous system, particularly the brain (Davis, Bushnell, Iannetti, St Lawrence, & Coghill, 2015). Within the last few decades cortical pain processing continues to be an ongoing and important topic in neuroscientific basic research. A 2005 meta‐analysis revealed that the commonest regions found to be active in pain processing are the S1 and secondary somatosensory cortex (S2), the anterior cingulate cortex (ACC), insular and prefrontal cortices, as well as the thalamus (Apkarian, Bushnell, Treede, & Zubieta, 2005). These regions represent a multimodal network, previously referred to as the ‘pain matrix’. However, recent research has shown that this so‐called ‘pain matrix’ is not exclusively pain specific as it is found to also be active in nonpainful sensory stimuli and especially involved in the detection of salient sensory inputs (Iannetti & Mouraux, 2010; Legrain, Iannetti, Plaghki, & Mouraux, 2011). This controversy depicts the current challenges in pain research and the ongoing search after neural correlates of (chronic) pain. Here, we were interested in the sensorimotor processing of painful stimuli. In the field of LBP research, several studies have applied various painful and nonpainful stimulations in order to extract the neural sensorimotor correlates of the pain experience and their possibly differential processing in patients compared to healthy controls; in primary somatosensory and motor cortices alterations were found mainly shifts or ‘smudging’ of representations (Flor, Braun, Elbert, & Birbaumer, 1997; Lloyd, Findlay, Roberts, & Nurmikko, 2008; Tsao, Galea, & Hodges, 2008; Wand et al., 2011). Despite these valuable insights in possibly altered cortical processes or reorganization in sensorimotor areas, evidence about central sensorimotor processing of LBP in healthy and especially in patients with CLBP remains sparse. Boendermaker, Meier, Luechinger, Humphreys, and Hotz‐Boendermaker (2014) applied an anterior‐to‐posterior nonpainful pressure stimulation on the spinous process of healthy subjects. As LBP is thought to be a mechanical disorder (Kobayashi et al., 2009), the application of a controlled and clinically relevant stimulus to the lower back is appropriate and important. However, motion artifacts restricted this functional magnetic resonance imaging (fMRI) experiment to the investigation of only nonpainful pressure stimuli (instead of painful stimulation) (Gervain et al., 2011; Meier, Hotz‐Boendermaker, Boendermaker, Luechinger, & Humphreys, 2014). Therefore, this study aimed at using functional near‐infrared spectroscopy (fNIRS) to measure cerebral hemodynamic responses, as fNIRS is more robust against motion artifacts as fMRI. FNIRS is a promising noninvasive optical method to study functional brain activity. Unlike other neuroimaging techniques, such as fMRI and positron emission tomography (PET), fNIRS is a portable and inexpensive method with high temporal resolution (Gervain et al., 2011; León‐Carrión & León‐Domínguez, 2012) that is also practicable for clinical bedside measurements. FNIRS enables the capturing of characteristic changes in oxyhemoglobin [O_2_Hb] and deoxyhemoglobin [HHb] that is elicited by brain activity due to neurovascular coupling (Ferrari & Quaresima, 2012; León‐Carrión & León‐Domínguez, 2012; Lloyd‐Fox, Blasi, & Elwell, 2010; Scholkmann, Kleiser, et al., 2014; Villringer & Chance, 1997). Near‐infrared light (NIR, i.e., within the spectral range of approx. 650–950 nm) penetrates skin, skull, and the brain relatively easily, and the intensity of the reemerging diffusely reflected light can be measured several centimeters apart from the light entry point (Delpy & Cope, 1997; Ferrari & Quaresima, 2012; Gervain et al., 2011; Lloyd‐Fox et al., 2010; Scholkmann, Kleiser, et al., 2014). The caveats of fNIRS are mainly its limited penetration depth, allowing measurements of approximately 1–3 cm into the cortex and its lower signal‐to‐noise ration (SNR) (Ferrari & Quaresima, 2012; Wolf et al., 2008).

In their study investigating cortical hemodynamic changes to nonpainful pressure on the lower back, Boendermaker et al. (2014) found robust bilateral activations in two sensorimotor areas, namely, the S1 and SMA, among other functional regions like the insula and anterior cingulate cortices. Both the SMA and the S1 have been shown to play an important role in the processing of sensorimotor inputs from the back, including also nociception and pain intensity coding (Boendermaker et al., 2014; Bushnell et al., 1999; Coghill, Sang, Maisog, & Iadarola, 1999; Peyron, Laurent, & Garcia‐Larrea, 2000; Xie, Huo, & Tang, 2009). Regarding the S1 area, recent work shows promising results for pain‐specific hemodynamic changes assessed by fNIRS during noxious and innocuous stimulation of the thumb in healthy subjects (Yucel et al., 2015). In addition, Uceyler et al. (2015) reported enhanced cortical activity in the S1 in patients with fibromyalgia syndrome after painful pressure stimulation at the dorsal forearm. However, the lower back has not yet been a target for combined painful pressure stimulation and cortical hemodynamic changes measurements by means of fNIRS. Therefore, this study intended to combine these two methodologies in order to enable painful pressure stimulation of the lower back. The regions of interest were chosen according to the study of Boendermaker et al. (2014), taking both sensorimotor regions which were showing robust activity during non‐painful pressure; the S1 and SMA. Both regions are located at the cortical surface and could be therefore probed by fNIRS.

To summarize, this study aimed at investigating (1) whether cortical activation in the S1 and the SMA due to different PA pressure on the lumbar spine can be detected using fNIRS; (2) whether it is possible to apply painful stimuli when measuring cortical responses by mean of fNIRS (instead of fMRI) to avoid motion artifacts, and (3) if it is possible to distinguish between painful and nonpainful pressure as well as a tactile stimulation (via a brushing stimulus) based on relative changes in [O_2_Hb] and [HHb] in both cortical regions. It is envisaged that this study should serve as a foundation for further fNIRS investigations in chronic low back pain patients. This approach may serve as a promising basis for further investigations regarding differential hemodynamic processing associated with neuroplastic changes in patients with CLBP.

## 2 Materials and Methods

### 2.1 Subjects

Twenty‐two healthy adult subjects participated in this study and twenty subjects (age: 33.5.7 ± 10.7 years, range: 22–61 years, 8 women) were included in the final data analysis. Two female subjects (both with long, dark, and curly hair) were excluded due to very poor fNIRS signal quality during the measurements. All subjects did not have a history of neurological disorders or chronic pain states before. Recruitment was done via online advertisement and word‐of‐mouth recommendation. Subjects were financially compensated for their participation.

The study was approved by the Ethics Committee of the Canton of Zurich (KEK‐ZH‐Nr. 2012–0029) and conducted in accordance with the Declaration of Helsinki.

### 2.2 Functional near‐infrared spectroscopy instrumentation

**Figure 1:**
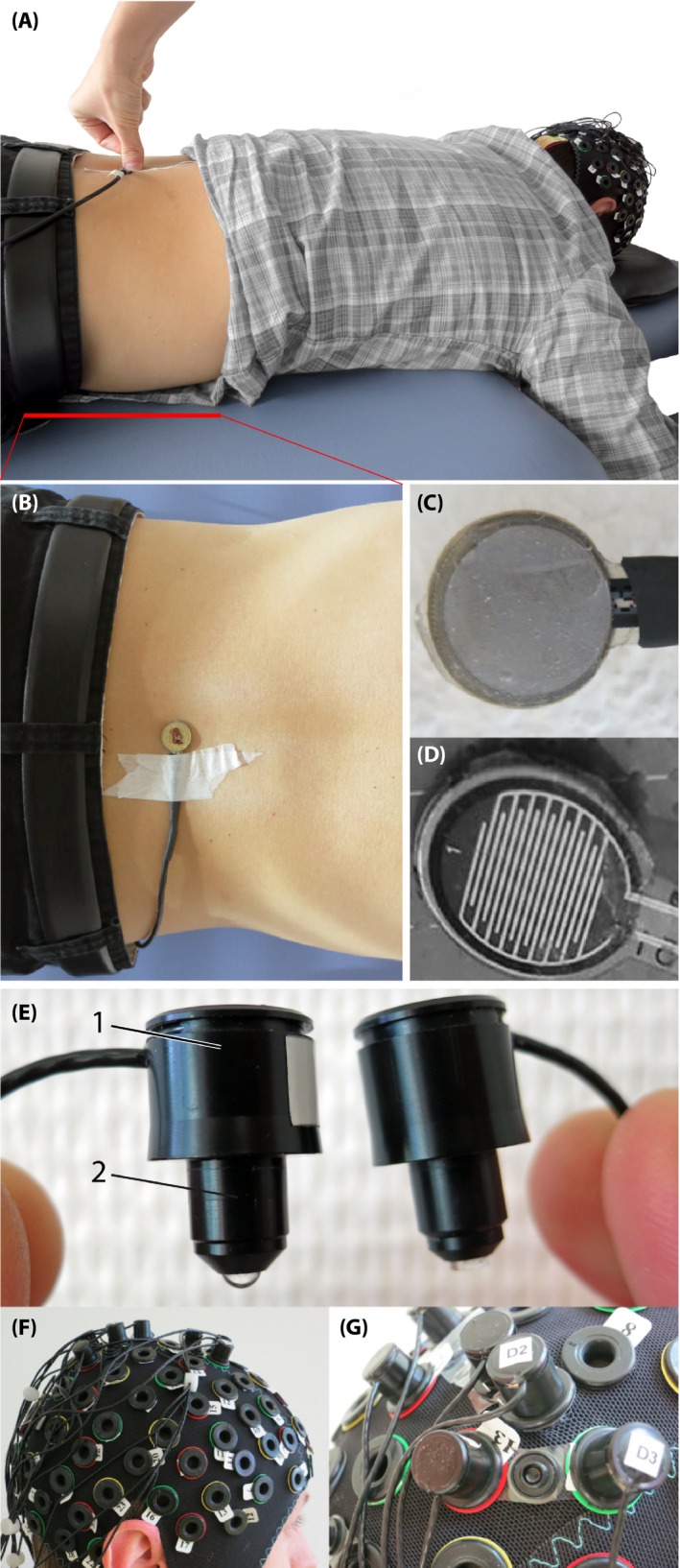
Experimental setup. (A) Subject lying in prone position on a massage bench, while the examinant is applying a posterior‐to‐anterior pressure on the force sensor attached on third lumbar *spinous process* (L3). (B) Pressure sensor from top view, attached on the L3. (C) The force sensor from the bottom (this side is placed on the skin), (D) The inside of the force sensor. (E) The optodes, left with 1) the enclosure of the optic fibers and 2) the tip of the light emission diode (LED) source and right of the detector. (F) Probe array on the subjects head, (G) Fixation of a source and detector distance by using so‐called distance holders

**Figure 2:**
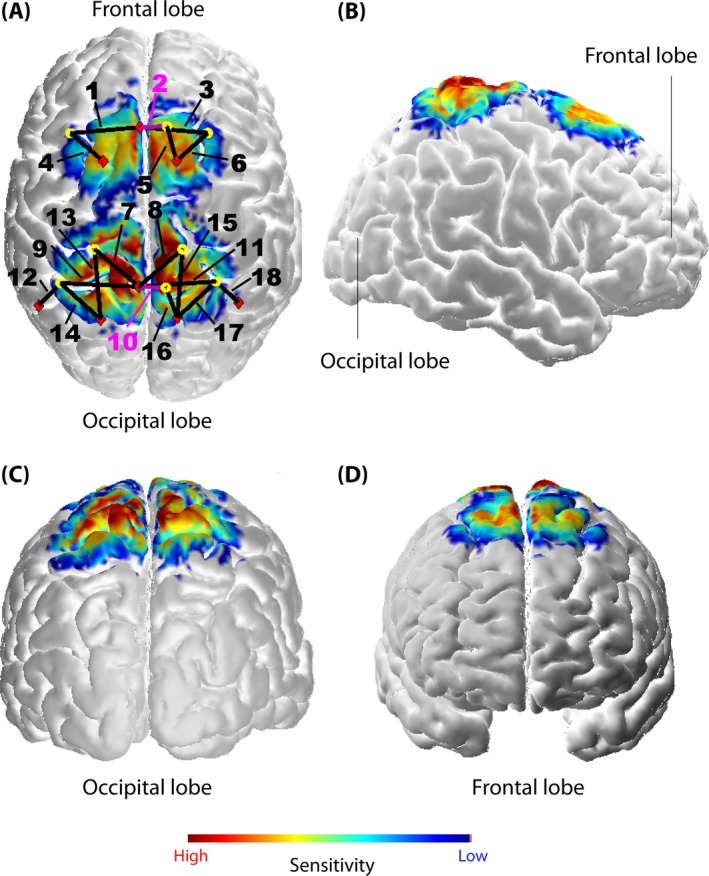
Probe array (A) and its sensitivity profile [per mm] (calculated using AtlasViewer as implemented in HOMER2) from different views (A–D). The probe array displays the sources (red rhombi), the detectors (yellow circles), and the channels (black lines, numbered; the two short‐distance channels are marked with pink lines and numbers). The sensitivity values are displayed in log10 units. The profile was calculated for 10 million photons

A multichannel continuous‐wave functional near‐infrared spectroscopy (fNIRS) imaging system (see Fig. 1E–G) (NIRSport, NIRx Medical Technologies LLC, NY, USA; RRID:SCR_014541; S/N: 1230/0001) operating at 760 nm and 850 nm was employed. The NIRStar Software 14.0 (NIRx Medical Technologies LLC; RRID:SCR_014540) was used for data recording. The probe contained 8 sources and 8 detectors, forming 18 multidistant channels (see Fig. 2A).

Signals were recorded with a sampling rate of 7.81 Hz. Channels covered two ROIs, that is, the S1 around the midline, enclosing back and trunk representation (Eickhoff, Grefkes, Fink, & Zilles, 2008), and the SMA according to the craniocerebral topography within the international 10–20 system as it was done previously by Wang et al. (2007) (Steinmetz et al., 1989). Channels 2 and 10 were short‐separation channels (with a source‐detector separation of ~11 mm). All other channels had a source‐detector distance ranging from 25 mm up to 45 mm. Multiple source‐detector distances were chosen in order to acquire hemodynamic responses from several locations within the ROIs. Particularly, the S1 within the postcentral gyrus comprises different Brodmann areas (BA 1, BA 2, BA 3a and 3b) which are not located at the same pitch of the gyrus (Geyer, Schormann, Mohlberg, & Zilles, 2000; Grefkes, Geyer, Schormann, Roland, & Zilles, 2001). Textile EEG caps (EASYCAP, Herrsching, Germany) in three different sizes (i.e., having a circumference of 54, 56, and 58 cm) were provided for the system to fix sources and detectors on the head. Anatomical differences between subjects’ heads had of course an impact on the exact separation of each channel. However, the probe arrangement was fixed in each of the caps in order to assure comparable probe placement over all subjects (see Fig. 1G). Additionally, a spatial sensitivity profile based on the Monte Carlo photon migration forward modeling was calculated using the AtlasViewer software (HOMER2 software package, http://www.nitrc.org/projects/homer2; RRID:SCR_009586) (Aasted et al., 2015) in order to assure that the specific probe placement enabled proper measurement of the ROIs (see Fig. 2A–D). Monte Carlo photon migration modeling was calculated for 10 million photons. The simulation revealed that the used probe setup is able to ensure that the fNIRS signals measured were also due to changes in the cerebral compartment of both ROIs.

### 2.3 Heart rate measurement

As an additional measurement, heart rate was assessed by employing a Garmin Edge500 device (Garmin Ltd., Schaffhausen, Switzerland; sampling rate 1 Hz) and a heart rate belt which was positioned at the lower sternum for the duration of the experiment.

### 2.4 Experimental design

Prior to the experiment, subjects had to fill out the German version of the Edinburgh Handedness Inventory (Oldfield, 1971). For the identification/assignment of the third lumbar vertebrae (L3), subjects were asked to stand straight with the back to the examiner. The L3 was manually palpated by the experienced examiner and marked. Head circumference was measured to assure adequate cap size. Subjects were seated on a chair while the cap was placed on their scalp. To assure proper positioning the nasion–inion length as well as the ear‐to‐ear distance were measured and the position of the cap was adapted according to the international 10–20 positioning system (Chatrian, Lettich, & Nelson, 1985). To ensure a good light coupling of the sensors/detectors, hair was brushed away within every hole of the cap and a clear ultrasound gel (Aquasonic clear ultrasound gel, PARKER Laboratories, INC.) was administered on the scalp (to keep the hair away) and the optodes were fixed.

For the experiment, subjects were lying in prone position on a massage bench. The whole experiment lasted 20 min. After a baseline measurement of 5 min, three different stimuli were applied in a pseudo‐randomized order (no more than two consecutive identical stimuli). Each stimulus was applied 15 times and the stimulus duration was 5 s. The interstimulus interval (ISI) was 15 s. Rounds of stimulus and ISI duration were manually clocked for the heart rate measurement. Stimuli consisted of painful and nonpainful mechanical pressure exerted with a thumb grip over a small circular plate, perpendicularly to the *spinous process* of the L3 (see Fig. 1A–B). The stimulation induced a posterior‐to‐anterior (i.e., dorso‐ventral) intervertebral movement (i.e., a PA‐pressure stimulus). This technique is a commonly used manual technique for assessment of spinal movement (joint play) and spinal treatment (Snodgrass, Rivett, & Robertson, 2006) in chiropractic therapy or in physiotherapy. In order to control for equal pressure forces, a force sensor (FlexiForce^®^Sensors, Teksan) was attached at the *spinous process* of the L3 (see Fig. 1B–D). The sensor included an amplifier that transformed the resistive changes in an appropriate voltage signal. The signal was digitalized by a microcontroller (1 KHz) and sent to a laptop where it was visible for the examinant throughout the experiment. The systematic error of this sensor was 10% of the applied force. One pressure stimulus was a nonpainful pressure force with 30 N with a standard deviation of ± 3 N (painfulness 0/10 on a visual analog scale [VAS]). The other was an individual painful pressure force which was determined prior to the experiment by identifying the individual pressure pain threshold (PPT) (Petzke, Harris, Williams, Clauw, & Gracely, 2005; Rainville, Feine, Bushnell, & Duncan, 1992). The PPT was assessed by slowly increasing pressure force on the L3 until the subject informed the investigator that the pressure reached the PPT and was clearly sensed to be painful (painfulness 3/10 on a VAS). This procedure was repeated 3–4 times and the obtained values were averaged for the final PPT, please see Table 1 for the detailed values. The third stimulus was a brushing (also by the examinant's thumb) over the left *musculus erector spinae*. The brushing was representing a different tactile stimulation type.

**Table 1:** Pressure pain thresholds (PPT) for each subject in Newton [N]

Pressure forces	
	PPT [N]
Subject 01	55
Subject 02	52
Subject 03	53
Subject 04	52
Subject 05	55
Subject 06	43
Subject 07	45
Subject 08	55
Subject 09	55
Subject 10	55
Subject 11	65
Subject 12	60
Subject 13	50
Subject 14	60
Subject 15	65
Subject 16	65
Subject 17	70
Subject 18	70
Subject 19	60
Subject 20	60

Thus, the experimental protocol consisted of applying three types of stimulations: (1) a nonpainful PA‐pressure stimulus with a force of ~30 N (PA30), (2) a painful PA‐pressure stimulus (PAPain), and (3) a stimulus comprising a brushing (Brush).

All stimuli were performed once before the experiment in all subjects to familiarize them with the experimental conditions. Prior to the start, subjects were advised to keep their eyes open during the whole experiment and to avoid moving. An easy cognitive task was imposed on the subjects to prevent them from falling asleep. They had to count the quantity of one of the three stimuli (they could freely choose which one they wanted to count). After the experiment the subjects had to report their counting results. Subjects being off the mark by more than two points would have been excluded from the analysis.

### 2.5 Data analysis and statistics

#### 2.5.1 fNIRS signal processing

Raw optical density (OD) data were first uploaded to the nirsLAB analysis software (NIRx Medical Technologies LLC), together with the probe information. By applying the modified Beer‐Lambert Law (Cope et al., 1988), the OD data were converted into the relative concentration changes in [O_2_Hb] and [HHb]. For each subject the age‐dependent Differential Pathlength Factor (DPF) was calculated according to the equation given by Scholkmann and Wolf (2013). For further analysis, datasets were exported from the nirsLAB software to Matlab (Version 2013b, Mathworks, Natick, MA, USA; RRID:SCR_001622). The datasets were band‐pass filtered applying the following process: high‐frequency noise of the signal was removed by applying a third degree Savitzky and Golay (1964) filter with a window length of 4 s (Schafer, 2011); the low‐frequency trend was removed by subtracting the low‐frequency trend from the data determined by applying the SG with window length of 80 s. The window length's (i.e., 4 s and 80 s) were chosen empirically in order to get the most robust hemodynamic response. Using the SG filter (instead of a simple moving average filter or a FIR/IIR filter) ensured that the filtering procedure preserves the most important information of the signals while removing the component, nonrelated to evoked hemodynamic changes (Savitzky & Golay, 1964; Schafer, 2011). After the filtering process the datasets were segmented into intervals with a length of 5 s (stimulus duration) plus 3.9 s of pre‐ISI and 3.9 s post‐ISI. Following this procedure the whole dataset was segmented into 15 intervals per condition. These segments were then detrended by applying a linear regression to remove the slow physiological drift during each segment period. Furthermore, the slices were normalized by subtracting the median value of the 3.9 s long pre‐ISI from the signal in each segment in order to remove the intraindividual variance of the starting values. To overcome the contamination of the fNIRS signal by hemodynamic changes happening in the superficial layers of the head (i.e., the scalp blood flow), a short separation regression (SSR) has been applied employing the approach presented by Saager and Berger (2005). The corrected [O_2_Hb] and [HHb] signals are determined with this approach by removing a weighted short‐channel signal from each long‐channel signal. The weighting factor is therefore determined by a least‐squares approach. For a concise summary of this method please refer to section 2.1.6 in Scholkmann, Metz, and Wolf (2014). For the SSR channels 2 and 10 (with a source‐detector separation of ~11 mm) were used. Channels 1 and 3–6 were corrected with SSR using channel 2, and channels 7–9 and 11–18 were corrected by SSR using channel 10.

Subsequently, in each single trial the median per sample was identified, resulting in a block average per subject, channel, and condition (PAPain, PA30, Brush). Furthermore, the grand averages per condition and group were calculated by taking again the median (from the middle 2.5 s of the 5 s lasting stimuli) of all the subjects. In order to assess variance of the datasets, the standard error of the median (SEMed) was calculated as well.

#### 2.5.2 Statistical analysis

As the data were generally not normally distributed the whole statistical analyses of the hemodynamic changes was conducted by using nonparametric tests. To test for statistical significance at single‐subject level, a Wilcoxon signed‐rank test was applied per channel to check for the null hypothesis that the median values have a distribution not different from zero. For the group‐level analyses, two different approaches were applied. The first (classic) approach (‘Analysis_All’) comprised all channels of all subjects, independently of which channels showed statistical significance in the previously calculated Wilcoxon signed‐rank test (at single‐subject level). The second approach (‘Analysis_Responders’) comprised only those channels into the group analysis which were classified being ‘responders’. Therefore, all channels exhibiting a task‐related hemodynamic change were included, while all channels not exhibiting any task‐related response were excluded from the group analyses. This approach decreases the rate of false negatives in the group analysis and takes into account that single subjects do not show significant hemodynamic responses. Both approaches were applied throughout this work for several statistical tests. First, the main effect of group was calculated by using a Friedman test. Results were corrected for multiple comparisons by the false discovery rate (FDR) correction following the Benjamini and Hochberg (1995) procedure implemented in Matlab (*q *<* *0.05). To test whether the three different conditions elicited characteristic hemodynamic responses per condition, grand averages (averaged medians) were calculated per each condition. Subsequently, Wilcoxon signed‐rank tests were applied per channel in order to compare the hemodynamic response against zero. Data were again corrected for multiple comparisons (FDR, *q *<* *0.05). Finally, post hoc paired Wilcoxon rank‐sum tests were computed to assess significant differences in the hemodynamic response among the three different conditions (FDR corrected, *q *<* *0.05).

#### 2.5.3 Analysis of habituation versus sensitization

Finally, a habituation and/or sensitization analysis was performed by using individual linear regressions. For each single subject, condition, and channel, one linear regression model was calculated to assess whether subjects show a sensitization (i.e., stronger cortical hemodynamic response to the stimulus) or habituation (i.e., weaker cortical hemodynamic response to the stimulus) over time.

#### 2.5.4 Heart rate analysis

For the heart rate analysis, averaged (*HR*_mean_) and maximal values (*HR*_max_) per stimulus (5 s) and per ISI (15 s) were extracted from the Garmin Edge500 device and exported to Excel (Microsoft, Redmon, WA, USA). In the case of the ISI there is a need for differentiating between pre‐ISI (the one before a specific stimulus) and the post‐ISI (the one after a specific stimulus). Moreover, for each condition (always per stimulus and pre‐ and post‐ISI) the mean, the standard deviation (SD), and the standard error of the mean (SEM) of *HR*_mean_ as well as *HR*_max_ were calculated for all subjects. Three paired Wilcoxon‐tests were used to calculate whether there was a significant difference between *HR*_max_ during the stimulus and *HR*_max_ during the post‐ISI (e.g., PAPain vs. post‐ISI pain) (*q *<* *0.05, FDR corrected). To answer the question whether there was a significant difference regarding heart rate between the different stimuli, the difference between the *HR*_max_ for the post‐ISI and *HR*_max_ during the stimulus was calculated and used for a paired Friedman test (FDR corrected, *q *<* *0.05).

## 3 Results

### 3.1 Stimulus‐evoked hemodynamic responses

Wilcoxon signed‐rank tests on single‐subject level revealed that 73% of the channels showed significant task‐related changes in [O_2_Hb] in the painful condition, 62% in the non‐painful condition, and 16% in the brushing condition. For [HHb] the success rate was slightly lower with 50% for the painful condition, 55% for the non‐painful, and 16% for the brushing condition.

At the group level, the Friedman test yielded at least three or more channels that significantly distinguished between the three conditions (for detailed results please see Table 2[a–b]) for both analysis approaches (*Analysis_All* and *Analysis_Responders*) for [O_2_Hb], whereas for [HHb] only uncorrected results (i.e., without correcting for the multiple‐comparison situation employing the FDR approach) showed significant differences for the three conditions in a few channels. These channels corresponded to the significant channels for [O_2_Hb], with the exception of channel 1.

**Table 2:** A+B: Main effect of condition for A) oxyhemoglobin ([O_2_Hb]) and B) deoxyhemoglobin ([HHb]). Channels 1–6 belong to the bilateral supplementary motor area (SMA), whereas channels 7–18 are belonging to the bilateral primary somatosensory cortex (S1). The subscript ‚ All’ means that all responders and nonresponders were included for analysis, whereas in the ‚ responders’ group a preselection took place. Shown are significant channels as well as tendencies, both corrected for multiple comparisons by applying false discovery rate (FDR) correction (q < 0.05)

A							
	Channel no.	[O_2_Hb]_All_			[O_2_Hb]_Responders_		
		*p* uncorrected	*q* (FDR)	χ^2^	*p* uncorrected	*q* (FDR)	χ^2^
SMA	1						
	3						
	4				.018	.0304	8.042
	5	.001	.016	13.3	.003	.016	11.645
	6	.004	.02	11.1	.015	.03	8.444
S1	7				.047		6.136
	8						
	9	.024		7.5	.019	.0304	7.875
	11	.043		6.3	.010	.03	9.220
	12	.015	.048	8.4	.000	.000	15.460
	13						
	14	.035	.08	6.7	.013	.03	8.667
	15				.033	.048	6.821
	16				.014	.03	8.522
	17	.005	.02	10.8	.000	.000	15.216
	18	.002	.016	12.1	.009	.03	9.418

B							
Region	Channel no.	[HHb]_All_			[HHb]_Responders_		
		*p* uncorrected	*q* (FDR)	χ^2^	*p* uncorrected	*q* (FDR)	χ^2^
SMA	1	.004		11.1	.033		6.837
	3						
	4						
	5				.006		10.383
	6						
S1	7						
	8						
	9	.024		7.5			
	11						
	12						
	13						
	14						
	15						
	16						
	17						
	18				.017		8.167

**Figure 3:**
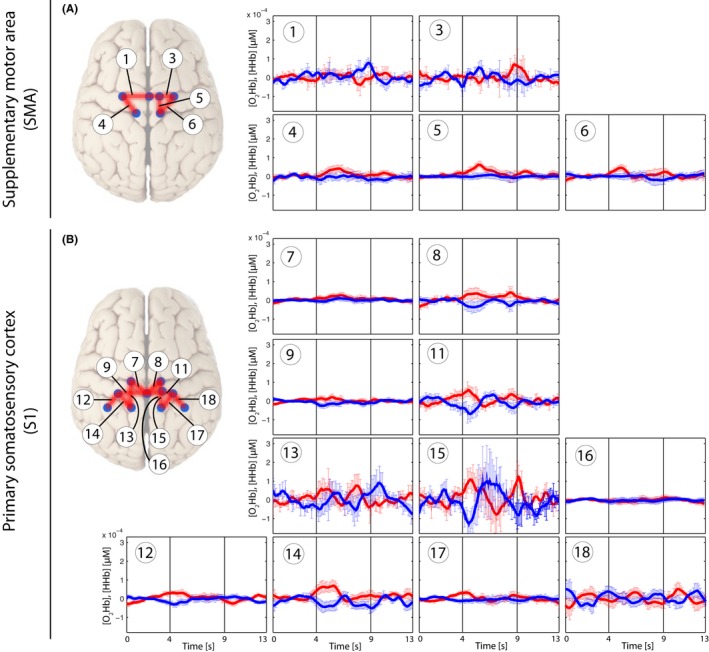
Grand average of evoked hemodynamic changes in all subjects *(Analysis_All)* due to the brushing stimulus on the SMA (A) and S1 (B). Changes in [O_2_Hb] (red) and [HHb] (blue) are depicted as changes in the median concentration. The two vertical lines within the graph represent the stimulus on‐ and offset (duration = 5 s). Error bars represent the standard error of the median

**Figure 4:**
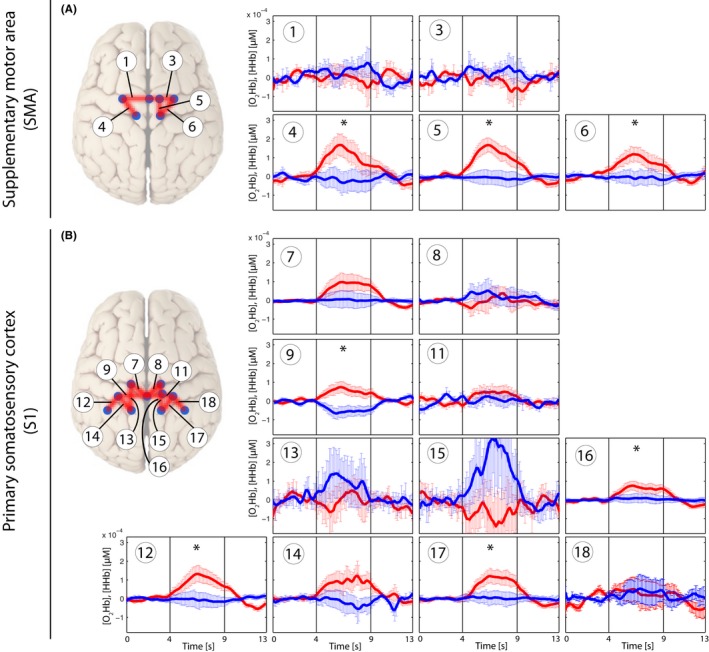
Grand average of evoked hemodynamic changes in all subjects *(Analysis_All)* due to the nonpainful PA‐pressure stimulus (PA30) on the SMA (A) and S1 (B). Changes in [O_2_Hb] (red) and [HHb] (blue) are depicted as changes in the median concentration. The two vertical lines within the graph represent the stimulus on‐ and offset (duration = 5 s). Error bars represent the standard error of the median. Significant changes (*q *<* *0.05; corrected for multiple comparison via [FDR]) are marked with a “*”

**Figure 5:**
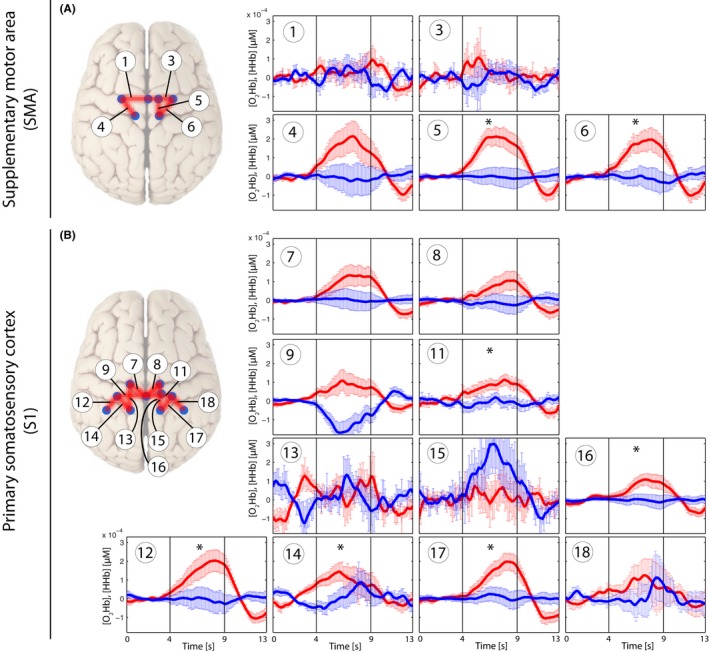
Grand average of evoked hemodynamic changes in all subjects (*Analysis_All*) due to the painful PA‐pressure stimulus (PAPain) on the SMA (A) and S1 (B). Changes in [O_2_Hb] (red) and [HHb] (blue) are depicted as changes in the median concentration. The two vertical lines within the graph represent the stimulus on‐ and offset (duration = 5 s). Error bars represent the standard error of the median. Significant changes (*q *<* *0.05; corrected for multiple comparison [via FDR]) are marked with a “*”

**Figure 6:**
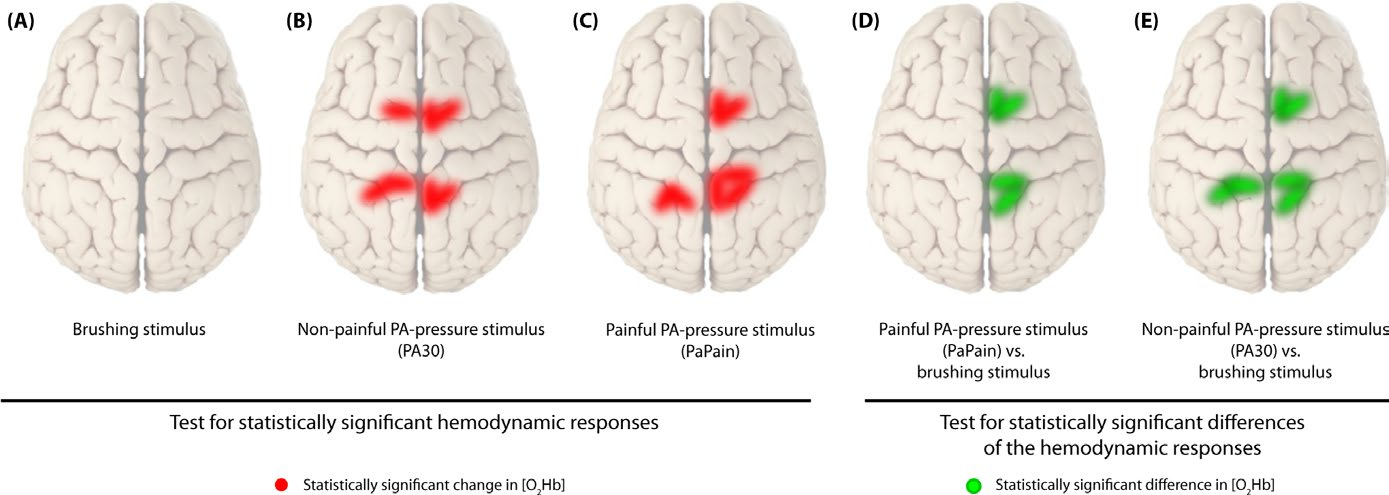
Visualization of statistically significant channels in different comparisons for [O_2_Hb]. (A) Brushing stimulus versus baseline, (B) Nonpainful posterior‐to‐anterior (PA) stimulus (PA30) versus baseline, (C) Painful PA‐pressure stimulus (PAPain) versus baseline, (D) PAPain versus the brushing stimulus, and finally, (E) PA30 versus the brushing stimulus. Therefore, red lines display statistically significant channels in comparisons versus baseline, whereas green lines display significant channels in comparisons of the conditions against each other (q < 0.05, corrected for multiple comparisons using false discovery rate [FDR])

Grand averages per condition are shown in Figs 3, 4, 5, 6 in order to visualize the dynamics of the hemodynamic responses and their significance (*Analysis_All* approach; for results of the Analysis_Responders approach, please see in the supporting part of this study).

Finally, post hoc paired Wilcoxon rank‐sum tests showed that both pressure conditions induced similar [O_2_Hb] changes in contrast to the brushing condition (Table 3). Furthermore, painful pressure provoked small differences compared to nonpainful pressure in [O_2_Hb] in a few channels (Table 3). However, these changes did not survive the FDR correction. [HHb] yielded only a few uncorrected changes in all three conditions shown in Table 4.

**Table 3:** Post hoc comparisons of relative oxyhemoglobin ([O_2_Hb]) changes between painful pressure (=PAIN), nonpainful pressure (=PA30), and brushing (=Brush). A) PAIN versus PA30, B) PAIN versus Brush, and C) PA30 versus Brush. The subscript ‚ *All*’ means that all responders and non‐responders were included for analysis, whereas in the ‚ *Responders*’ group a pre‐selection took place. Results are false discovery rate (FDR) corrected (*q *<* *0.05)

Comparison	Channel	[O_2_Hb]_All_			[O_2_Hb]_Responders_		
		*p*_uncorrected_	*q* (FDR)	*Z*	*p*_uncorrected_	*q* (FDR)	*Z*
A) PAIN vs. PA30	1						
	3						
	4						
	5	.019		−2.352			
	6	.037		−2.091			
	7						
	8						
	9				.028		−2.201
	11						
	12						
	13						
	14	.008		−2.651			
	15				.050		−1.956
	16				.015		−2.432
	17						
	18	.021		−2.315			
B) PAIN vs. Brush	1						
	3						
	4				.028		−2.197
	5	.002	.0107	−3.024	.002	.0213	−3.051
	6	.002	.0107	−3.136	.016		−2.411
	7				.048		−1.977
	8						
	9	.023		−2.277	.034		−2.118
	11	.006	.0240	−2.725	.013		−2.497
	12	.028		−2.203	.004	.0213	−2.844
	13						
	14	.040		−2.053	.044		−2.017
	15						
	16	.021		−2.315	.023		−2.275
	17	.002	.0107	−3.061	.003	.0213	−2.934
	18						
C) PA30 vs. Brush	1						
	3						
	4				.017		−2.395
	5	.007	.04	−2.688	.004		−2.900
	6	.015	.04	−2.427	.021		−2.312
	7				.031		−2.158
	8						
	9	.009	.04	−2.613			
	11	.014	.04	−2.464	.018		−2.366
	12	.012	.04	−2.501	.011		−2.551
	13						
	14						
	15						
	16	.037		−2.091			
	17	.001	.016	−3.360	.011		−2.551
	18				.023		−2.271

**Table 4:** Post hoc comparisons of relative deoxyhemoglobin ([HHb]) changes among painful pressure (=PAIN), nonpainful pressure (=PA30), and brushing (=Brush). A) PAIN versus PA30, B) PAIN versus Brush, and C) PA30 versus Brush. The subscript ‚ All’ means that all responders and nonresponders were included for analysis, whereas in the ‚ Responders’ group a preselection took place

Comparison	Channel no.	[HHb]_All_			[HHb]_Responders_		
		*p*_uncorrected_	*q* (FDR)	*Z*	*p*_uncorrected_	*q* (FDR)	*Z*
A) PAIN vs. PA30	1	.057		−1.904			
	3						
	4						
	5				.028		−2.197
	6						
	7				.041		−2.040
	8				.046		−1.992
	9						
	11						
	12						
	13						
	14	.040		−2.053	.039		−2.062
	15						
	16	.017		−2.389			
	17						
	18	.004		−2.912			
							
B) PAIN vs. Brush	1	.067		−1.829			
	3						
	4						
	5						
	6						
	7						
	8						
	9						
	11						
	12						
	13						
	14						
	15						
	16						
	17						
	18	.093		−1.680			
C) PA30 vs. Brush	1						
	3						
	4						
	5						
	6	.057		−1.904			
	7						
	8						
	9	.030		−2.165			
	11						
	12						
	13						
	14						
	15				.075		−1.782
	16						
	17						
	18	.048		−1.979			

FDR, false discovery rate.

**Figure 7:**
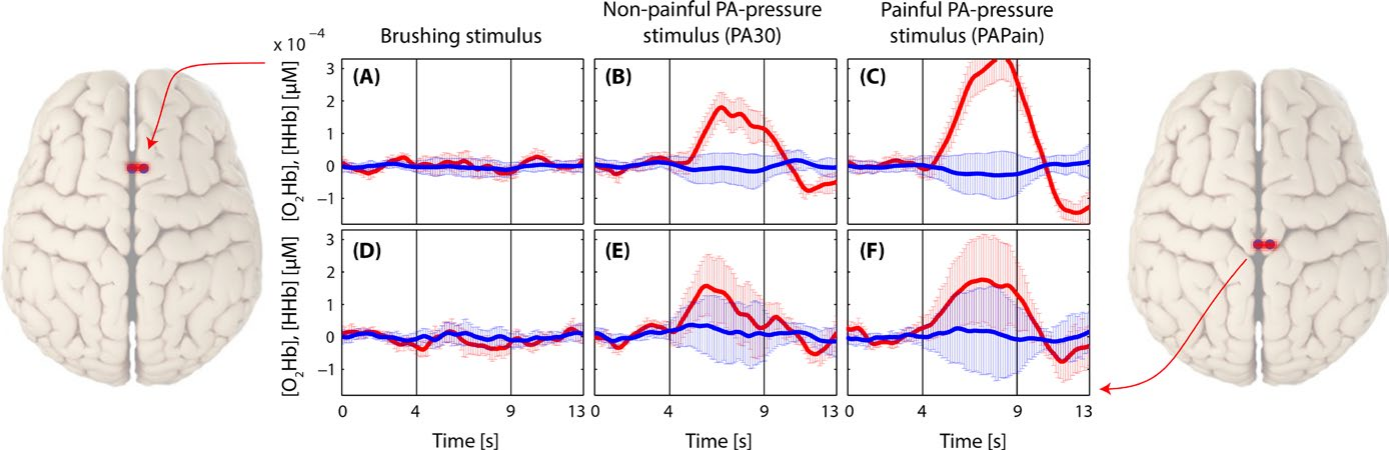
Grand average of evoked hemodynamic changes in all three conditions in the two short‐distance channels (channels 2 and 10) before the application of the short separation regression. (A–C): Short‐distance channel in the supplementary motor area (SMA) in the brushing condition (A), in the nonpainful condition (B), and in the painful condition (C). (D–F): Short‐distance channel in the primary somatosensory cortex (S1) in the brushing condition (D), in the nonpainful condition (E), and in the painful condition (F)

In addition, the grand averages of the two short‐distance channels for all three conditions before the regression are shown in Fig. 7, revealing that painful pressure induced the highest extracerebral task‐related systemic effect compared to the other conditions.

### 3.2 Habituation versus sensitization

Individual linear regression models at the single‐subject level revealed several significant correlations in a couple of [O_2_Hb] channels (*p *<* *.05). Eight subjects showed positive correlations in maximally 1–3 channels per subject. Five subjects showed negative correlations in maximally 1–3 channels. Therefore, both cortical habituation and sensitization could be detected in a few channels in some subjects, however, no group correlation or at least specific correlations for specific channels could be found.

### 3.3 Heart rate changes

**Figure 8:**
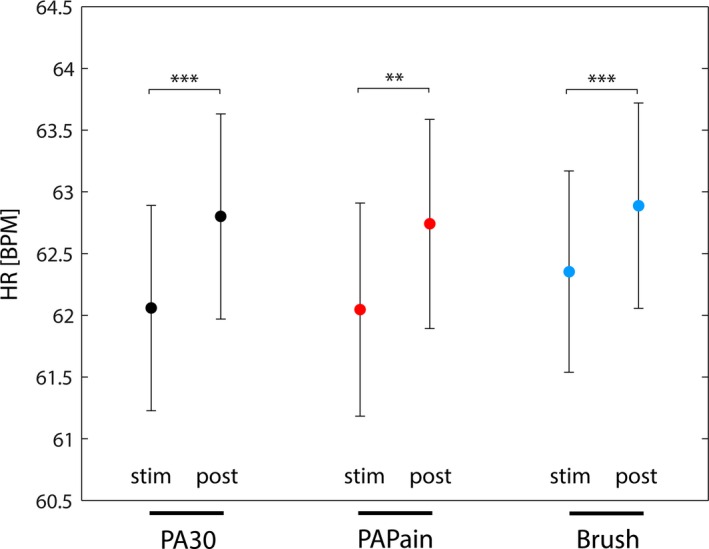
The average maximal heart rate (*HR*_max_) of all subjects during the stimuli and during the postinterstimulus intervals (post‐ISI). A statistical comparison was calculated by using Wilcoxon paired tests, yielding significant differences between the *HR*_max_ of the stimulus and the *HR*_max_ of the post‐ISI. Significance is indicated by stars (**: *p* < .002; ***: *p* < .001; corrected for multiple comparison [via FDR])

*HR*_max_ analysis revealed on the one hand a clear effect of condition as the post‐ISI *HR*_max_ was found to be significantly higher than the *HR*_max_ during the stimulus itself in all three conditions (*q *<* *0.05, FDR‐corrected) (see Fig. 8). On the other hand, no statistically significant difference in *HR*_max_ between the three conditions could be observed.

## 4 Discussion

This study aimed at investigating neural correlates of painful and nonpainful pressure stimulations on the lower back of healthy subjects measured by fNIRS. The results demonstrate that fNIRS is a feasible optical neuroimaging method to measure [O_2_Hb] and [HHb] changes in cortical sensorimotor areas due to mechanosensory stimulations on the lower back. Robust activity has been observed in S1 and SMA after both painful and nonpainful pressure, whereas brushing yielded no significant activity within these cortical areas.

### 4.1 Methodological aspects

#### 4.1.1 Pressure pain stimulation

To date, the majority of studies investigating neural correlates of pain in healthy subjects utilized thermal or electrical stimuli to provoke acute pain sensations (Apkarian et al., 2005). However, a pressure pain stimulus like the PA pressure represents a more relevant type of stimulus than an acute electrical or thermal pain stimulus, as low back pain is being thought to be a mechanical disorder (Kobayashi et al., 2009). A PA‐pressure stimulus on a *spinous process* represents a common diagnostic as well as therapeutic intervention in manual medicine (Snodgrass et al., 2006) and elicits motion within the so‐called functional spinal units (FSUs). The FSUs represent the smallest physiological motion entity of the spine. By applying a PA‐pressure stimulus to a FSU, especially mechanoreceptors (and nociceptors as well in the case of painful stimulation) are being activated, sending proprioceptive information on position, load, and motion to the central nervous system (CNS) (Schnitzler & Ploner, 2000; Tracey & Mantyh, 2007). As painful PA‐pressure stimuli presumably activate both superficial as well as deep mechanoreceptors and nociceptors, different (i.e., in parallel) ascending pathways of the nociceptive system may be engaged. Additionally, it has recently been suggested that by calculating a factor analyses of responses to diverse experimental pain modalities (e.g., pressure, heat, electrical) that different pain modalities may represent specific dimensions and different pathways (Hastie et al., 2005; Neziri et al., 2011) and hence may provoke different patterns of responses.

#### 4.1.2 Extracerebral task‐related effects

FNIRS measurements are highly influenced by task‐related or task‐unrelated changes, both of no interest, within the extracerebral compartment (e.g., skin, scalp, cerebrospinal fluid, and more). The study from Holper et al. (2014), who have investigated painful and nonpainful pressure stimulation at different sites of the lower back (provided by an algometer) and its effect on prefrontal activity using fNIRS, did show such an extracerebral confounding factor very nicely. They had measured the partial pressure of end‐tidal carbon dioxide (PetCO_2_) by capnography in order to quantify the effect of respiration on cortical hemodynamics. Even though they did not find characteristic [O_2_Hb] and [HHb] changes due to the different stimulations, they were able to reveal a strongly confounding effect of “respiration‐related‐changes in the partial pressure of CO_2_ in arterial blood (PaCO_2_) on fNIRS‐derived parameters” (Holper et al., 2014). Changes in PetCO_2_ can have significant impacts on fNIRS signals (Scholkmann, Gerber, Wolf, & Wolf, 2013; Scholkmann, Klein, Gerber, Wolf, & Wolf, 2014; Scholkmann, Wolf, & Wolf, 2013). A main reason for this confounding effect in the fNIRS signal might have been that they did not apply short separation regression within their analyses in order to get rid of confounding hemodynamics of the extracerebral layers (Gregg, White, Zeff, Berger, & Culver, 2010; Saager & Berger, 2005). In this investigation we implemented two short‐distance channels in order to measure superficial hemodynamics within each ROI. Confounding hemodynamic effects within the superficial layer of the head could therefore be reduced. Certainly, it would be desirable to use one short‐distance channel per one long‐distance channel, although the overall number of available channels of our device was limited. Nevertheless, already these two short‐distance channels enabled us to remove a substantial part of extracerebral task‐related systemic effects in both pressure conditions (see Fig. 7). In the brushing condition, the superficial effect was negligible, as it could be expected from the results comparing the conditions versus baseline (see Fig. 3). Moreover, the short‐distance channels display nicely (see Fig. 7) that painful pressure evoked higher extracerebral effects than non‐painful pressure and this yields evidence about how important such a correction is for accurate interpretation of fNIRS measurements. Additionally, it can be seen that [O_2_Hb] represents the main confounding factor, while superficial effects in [HHb] are quite small. This is in line with the current evidence from literature (Tachtsidis & Scholkmann, 2016). Interestingly, scalp blood flow changes (especially in the [O_2_Hb] signal) have been also observed during stimulations of sensorimotor areas due to nonpainful and painful electrical muscle stimulations (Muthalib et al., 2015). It could be shown that these task‐related scalp blood flow changes took place and that correcting for them is necessary.

In addition, we conducted heart rate measurements over the course of the experiment in order to monitor related physiologic responses. This parameter will be discussed in a later section below.

### 4.2 Stimulus‐evoked hemodynamic responses

First, the application of nonpainful pressure stimulation on the lumbar vertebra yielded a characteristic increase in [O_2_Hb] in several channels within both ROIs, whereas changes in [HHb] remained constant or decreased, although this decrease failed to reach significance after FDR correction. The bilateral S1 activation was located near by the midline, corresponding to the representation of the trunk in the human somatosensory homunculus (see Fig. 6) (Penfield & Boldrey, 1937; Rasmussen & Penfield, 1947). This result supported our expectations, as it is in agreement with the results of similar previous studies (Boendermaker et al., 2014; Kobayashi et al., 2009) who revealed robust bilateral S1 as well as SMA activity after applying nonpainful pressure stimulations on the lower back. However, the absence of significant changes in [HHb], especially when considering assumptions of hemodynamics underlying the fMRI signal, gives reason for discussion. Several investigations have already compared fNIRS data with the blood oxygen level‐dependent (BOLD) response derived from fMRI data. Nonetheless, results remain ambiguous. Some investigations (Huppert, Hoge, Diamond, Franceschini, & Boas, 2006; MacIntosh, Klassen, & Menon, 2003; Toronov et al., 2001) point to a better correlation between [HHb] and the BOLD response, as it might also be expected from the definition of the BOLD response (Ogawa, Lee, Kay, & Tank, 1990). However, a contradictory finding was published by Yamamoto and Kato (2002) as well as Strangman, Culver, Thompson, and Boas (2002) who found a better correlation of [O_2_Hb] and the BOLD response. Additionally, an investigation of motor tasks using fNIRS revealed that [O_2_Hb] might be a better indicator of CBF changes compared to the expected [HHb] (Anwar et al., 2013). Therefore, further research comparing signals from both modalities is needed in order to elucidate this relationship and also considering different cortical areas and stimulus modalities.

Second, the painful pressure stimulation yielded also characteristic increases in [O_2_Hb] in several channels within both ROIs, but significant channels were bilaterally distributed only within the S1, whereas within the SMA‐only channels on the right hemisphere yielded significance after FDR correction (see Fig. 6). Additionally, when comparing the painful versus the nonpainful condition, the painful condition induced slightly higher [O_2_Hb] responses than the nonpainful condition, although this difference was not significant after correction for multiple comparisons. Nevertheless, the current results are in line with other investigations. Yucel et al. (2015) revealed significant changes in the S1 in both [O_2_Hb] and [HHb] after noxious and innocuous electrical stimulation on the left thumb. Naturally, their detected S1 activity was observed to be in a more lateral area of the S1 representing the thumb. The S1 was reported afore as being among the commonest regions found to be active in pain processing, by a meta‐analysis of Apkarian et al. (2005). More specifically, it was suggested that the S1 resumes a central role in the sensory‐discriminative component of pain processing (Bushnell et al., 1999; Xie et al., 2009). However, in contrast to Yucel et al. (2015) the current results failed to reveal significant changes in [HHb]. This might be explained by different aspects. First, Yucel et al. (2015) described their electrical stimuli as innocuous and noxious stimuli. At this point, it is crucial to differentiate between nociception and pain perception. Nociception is the physiological process which can lead to pain perception, however, this is not necessary. The other way round, pain perception is a multimodal experience and does not exclusively underlie nociception, although often this is the case (Treede, 2006). According to their subjective rating of the stimuli, it can be assumed that the innocuous stimulus was already slightly painful (rating 3/10), whereas the noxious stimulus was rated much more painful (7/10). Nevertheless, they describe the innocuous stimulus as nonpainful. Certainly, comparing their subjective rating with the present ones, the painful PA ranged at the level of the PPT and the nonpainful stimulus was subjectively rated a 0/10, so truly not painful. Therefore, the overall intensity of the stimuli in the investigation of Yucel et al. (2015) was higher compared to the current one. As relative changes in both [O_2_Hb] and [HHb] proved to be higher after more painful stimulation in both studies, a lack of significance of the [HHb] changes in our study might be explained by too low a stimulation threshold. Second, although [HHb] has been revealed to show more selective and localized responses to hemodynamic changes (Cannestra, Wartenburger, Obrig, Villringer, & Toga, 2003), it also has been shown to reveal a lower sensitivity to changes in cortical activity due to its lower SNR ratio (Mihara, Miyai, Hatakenaka, Kubota, & Sakoda, 2008; Miyai et al., 2001). Additionally, [HHb] changes show generally much smaller changes compared to the large overshoot of [O_2_Hb] (Fox & Raichle, 1986; Tachtsidis & Scholkmann, 2016). Finally, comparing the statistical analysis, Yucel et al. (2015) did not correct for multiple comparisons (missing control for type I errors) (Singh & Dan, 2006) which might explain the missing significance of corrected [HHb] data in this study as well.

Furthermore, the activity of the SMA in response to the painful and nonpainful PAs is also in line with several former investigations (Coghill et al., 1999; Misra & Coombes, 2014; Peyron et al., 2000). However, its role in this context is not explored yet to that extent as the role of the S1. There is evidence that the SMA is involved in postural control, as it was shown by studies investigating anticipatory postural adjustments using fNIRS and transcranial magnetic stimulations (Jacobs, Lou, Kraakevik, & Horak, 2009; Mihara et al., 2008). Additionally, a major role is assigned to the SMA in motor planning, early motor preparation, and motor imagery (Hanakawa et al., 2003; Hetu et al., 2013; Iseki, Hanakawa, Shinozaki, Nankaku, & Fukuyama, 2008; Vrana et al., 2015; Wilson, Kurz, & Arpin, 2014). Based on this evidence, the activity of the SMA, might represent an early motor preparation as a response to both painful and nonpainful pressure on the lower back in order to stabilize the trunk against the ‘perturbation’ in terms of the pressure (Kobayashi et al., 2009; Schnitzler & Ploner, 2000). However, again [O_2_Hb] revealed to be the more sensitive parameter for cortical activation than [HHb], which did not survive FDR correction.

### 4.3 Statistical analysis

The statistical analyses in this work comprised two different approaches, the *Analysis_All* (including all channels no matter what the single‐subject analyses yielded) and the *Analysis_Responders*. These two approaches are representing distinct intentions. The classic *Analyses_All* approach, which is disregarding the single‐subject level, is including subjects and channels which are not showing any task‐related changes. Therefore, in this approach we include randomly noisy channels. This factor does influence the results, by allowing for more false‐negative results. Meanwhile, the *Analysis_Responders* approach is able to decrease the false‐negative rate by already excluding channels on single‐subject level which are showing random noise. Although this approach is not preselecting the data regarding quality, as, for example, systemic changes (large increases in both [O_2_Hb] and [HHb]) would also reach significance at single‐subject level and would be included into further group analyses.

Here, we are showing both analyses approaches, as in our opinion it adds value having both approaches of the same dataset. As it can be seen in Tables 2, 3, 4, there are no horrendous differences between the approaches, however, both analyses are supplementing each other. Particularly in detailed analyses like the post hoc Wilcoxon tests, searching for differences between the different conditions it is important that we know that we are calculating with task‐related hemodynamic changes and that those are not spoiled by noise.

There are advantages and disadvantages of both methods and we propose to implement both approaches in further fNIRS and probably also fMRI investigations in order to take into account the individual physiological response to a stimulus or task (Tachtsidis & Scholkmann, 2016) enabling an objective analyses from two distinct perspectives.

### 4.4 Habituation and sensitization

Both habituation and sensitization of the hemodynamic response could be detected in a few channels. However, results were too inconsistent to show a tendency at group level. This finding stands in contrast with the results of Yucel et al. (2015) who found a habituation effect in their noxious condition for the S1 region and no habituation effect in the innocuous condition. However, as mentioned before, the intensity difference between their noxious stimuli compared to our painful stimulus might explain this absence of a habituation effect in our experiment. Additionally, it has been shown that the duration of the ISI plays a major role in temporal summation of mechanically induced pain (Sarlani & Greenspan, 2002) and that the temporal summation decreases the longer the ISI is.

### 4.5 Heart rate

The heart rate analysis revealed an effect of condition compared to the ISIs. However, no difference was found between the heart rates during the different stimuli. Therefore, this physiological parameter did not reflect the difference between the stimuli. First, this result underlines the importance of the short separation regression analysis in order to control for hemodynamic changes (generally associated with an increased heart rate) within the superficial layers. Second, the missing difference between the three stimulations might indicate that the PPT was too ‘low’, in order to induce significantly different changes regarding heart rate than the other stimulations.

## 5 Limitations

This investigation reports on results about cortical hemodynamic changes in sensorimotor areas after painful and nonpainful stimuli. Nevertheless, there are some limitations to mention. Although the PA‐pressure stimulation technique is frequently applied in the clinical setting, its application in research is novel. Therefore, its use in neuroscience research is a big advantage over other pain modalities. Nevertheless, these manually applied stimulations cannot be as specifically administered as, for example, stimulations by laser optodes or electrodes. Therefore, a certain imprecision has to be taken into account, which should be improved by further development of this stimulus modality for research applications. A further limitation of the study is related to the method to remove extracerebral hemodynamic effects. Although the short‐separation regression was applied in order to remove hemodynamic changes in the superficial layer, thereby interfering with the cerebral responses, this could be improved by increasing the number of short‐distance channels. However, due to technical restrictions of our fNIRS imaging device, we were confined to implement only two short‐distance channels, one per ROI. By applying multiple short‐distance channels per ROI, the SNR could be further improved, allowing for a more exact insight into solely cerebral hemodynamics. Also the impact of systemic changes on the cerebral hemodynamics itself should be assessed by future studies in this area. This could be done, for example, by measuring PetCO_2_ in combination with fNIRS.

## 6 Conclusion and Outlook

To conclude, this investigation shows the feasibility of using fNIRS for the measurement of hemodynamic responses due to painful pressure stimulation on the lower back. Painful and nonpainful pressure yielded similar characteristic changes in mainly [O_2_Hb] in both SMA and S1, whereas the brushing stimulation failed to elicit characteristic hemodynamic changes. Moreover, [HHb] did not reveal significant (corrected) changes for all three stimulations. However, as fNIRS proved to be a feasible and reproducible optical imaging method, this investigation will serve as a foundation for further measurements in LBP patients in order to provide an insight to their neuronal processing of LBP.

## Conflicts of Interest

All authors declare no potential conflicts of interest with respect to the authorship and/or publication of this article.
