# Supplementary material for: Different mechanosensory stimulations of the lower back elicit specific changes in hemodynamics and oxygenation in cortical sensorimotor areas—A fNIRS study
Source: Brain Behav. 2016 Oct 14;6(12):e00575. doi: 10.1002/brb3.575 (PMC5167005; doi:10.1002/brb3.575)
Supplement: Supplementary file 1 [file BRB3-6-e00575-s001.docx]

| **Comparison** | **Channel no.** | **[O_2_Hb]_All_** | | **[O_2_Hb]_Responders_** | |
| --- | --- | --- | --- | --- | --- |
|  |  | *p_uncorrected_* | *q* (FDR) | *p_uncorrected_* | *q* (FDR) |
| PAIN  vs.  Baseline | 1 |  |  |  |  |
|  | 3 |  |  |  |  |
|  | 4 |  |  | 0.0161 | 0.0368 |
|  | 5 | 0.0004 | 0.0064 | 0.0061 | 0.0098 |
|  | 6 | 0.001 | 0.008 | 0.0081 | 0.0314 |
|  | 7 |  |  |  |  |
|  | 8 |  |  |  |  |
|  | 9 | 0.0333 |  | 0.0342 |  |
|  | 11 | 0.0152 | 0.0347 | 0.0098 | 0.0314 |
|  | 12 | 0.01 | 0.0347 | 0.0034 | 0.0208 |
|  | 13 |  |  |  |  |
|  | 14 | 0.0111 | 0.0347 | 0.0259 |  |
|  | 15 |  |  |  |  |
|  | 16 | 0.0137 | 0.0347 | 0.0122 | 0.0325 |
|  | 17 | 0.0040 | 0.0213 | 0.0039 | 0.0208 |
|  | 18 |  |  |  |  |
| PA30  vs.  Baseline | 1 |  |  |  |  |
|  | 3 |  |  |  |  |
|  | 4 | 0.0152 | 0.0347 | 0.0098 | 0.0499 |
|  | 5 | 0.00089 | 0.0142 | 0.0009765 | 0.0156 |
|  | 6 | 0.0072 | 0.0288 | 0.0137 | 0.0499 |
|  | 7 |  |  | 0.0353 |  |
|  | 8 |  |  |  |  |
|  | 9 | 0.0124 | 0.0347 |  |  |
|  | 11 | 0.0479 |  | 0.0156 | 0.0499 |
|  | 12 | 0.0152 | 0.0152 | 0.0134 | 0.0499 |
|  | 13 |  |  |  |  |
|  | 14 |  |  | 0.0494 |  |
|  | 15 |  |  |  |  |
|  | 16 | 0.0045 | 0.0288 |  |  |
|  | 17 | 0.0057 | 0.0288 | 0.0269 |  |
|  | 18 |  |  |  |  |
| Brush  vs.  Baseline | 1 |  |  |  |  |
|  | 3 |  |  |  |  |
|  | 4 |  |  |  |  |
|  | 5 |  |  |  |  |
|  | 6 |  |  |  |  |
|  | 7 |  |  |  |  |
|  | 8 |  |  |  |  |
|  | 9 |  |  |  |  |
|  | 11 |  |  |  |  |
|  | 12 |  |  |  |  |
|  | 13 |  |  |  |  |
|  | 14 |  |  |  |  |
|  | 15 |  |  |  |  |
|  | 16 |  |  |  |  |
|  | 17 |  |  |  |  |
|  | 18 |  |  |  |  |

**Table S1:** Comparisons of all three conditions against baseline for oxyhemoglobin ([O_2_Hb]). Deoxyhemoglobin ([HHb]) did not reveal any significance in both analyses (‘*All*’ and ‘*Responders*’). PAIN = painful pressure; PA30 = non-painful pressure and Brush = brushing.
